# Influence of Playing Site and Weekly Training Frequency on Physical Performance in Elite Padel Players

**DOI:** 10.3390/jfmk11010111

**Published:** 2026-03-06

**Authors:** Adrián González-Jiménez, Diego Muñoz, Adrián Castaño-Zambudio, Bernardino J. Sánchez-Alcaraz, Iván Martín-Miguel

**Affiliations:** 1 Sport Sciences Faculty, Rey Juan Carlos University, 28943 Madrid, Spain; adrigj1833@gmail.com (A.G.-J.); adrian.castano@urjc.es (A.C.-Z.); 2 Sport Sciences Faculty, University of Extremadura, 10003 Cáceres, Spain; diegomun@unex.es; 3 Sport Sciences Faculty, University of Murcia, 30730 San Javier, Spain; 4 Institute of Health and Sport Sciences, Faculty of Health Sciences, Universidad Francisco de Vitoria, 28223 Madrid, Spain; ivan.martinmiguel@ufv.es

**Keywords:** racket sports, high-level athletes, strength, vertical jump, maximum oxygen consumption, agility

## Abstract

**Objectives:** The physical and physiological characteristics of padel players are essential for appropriate training load prescription; however, this area remains underexplored. Therefore, this hypothesis-driven study aimed to analyse physical and physiological differences in male padel players according to playing side. A secondary objective was to observe the influence of training volume on these parameters. **Methods:** Fourteen high-level male players competing in professional circuits or top-level regional competitions participated in this cross-sectional study using directional (one-tailed) testing. **Results:** Vertical jump performance differed significantly between the countermovement jump (CMJ) and the Abalakov jump (ABK) (*p* < 0.001), with lower values in the CMJ (40.98 cm) compared with the ABK (46.96 cm). Isometric handgrip strength showed significant inter-limb differences (*p* < 0.001), with greater force in the dominant hand (49.08 kg) than in the non-dominant hand (44.22 kg). Mean completion time in the agility *t*-test was 10.40 s (95% CI: 10.06–10.74 s). The Yo-Yo Intermittent Recovery Test showed a mean distance of 404.28 m, corresponding to an estimated VO_2_max of 50.79 mL·kg^−1^·min^−1^. Playing side significantly affected Yo-Yo performance and estimated VO_2_max (*p* = 0.036), with higher values in left-side players. Although no significant differences were found in handgrip strength according to playing side. As expected, weekly training frequency did not significantly influence any variable. **Conclusions:** These findings help characterise the physical and physiological profile of high-level padel players and provide practical reference values to support training prescription and performance monitoring.

## 1. Introduction

Padel is a racket sport played in doubles on an enclosed 20 × 10 m court and is currently experiencing substantial worldwide growth [1]. Over the past decade, both its popularity and federated participation have increased markedly [2], driven by its competitive accessibility and high technical–tactical complexity [3]. In parallel with this expansion, the sport has evolved from a tactical perspective [4], leading to increased physical demands in modern padel [5]. Nevertheless, despite growing scientific interest and the practical relevance of these demands, research focusing on physical and physiological parameters remains limited when compared with the extensive literature addressing performance-related indicators [6].

From a physical and physiological standpoint, padel is characterized as a high-intensity intermittent sport, with an approximate stroke frequency of 0.78 shots·s^−1^ [7], involving the alternation of short- and medium-duration efforts (5–20 s) with intermittent recovery periods. These demands are associated with blood lactate concentrations ranging from 2.40 to 3.38 mmol·L^−1^ [8], as well as a mean oxygen uptake of approximately 24 mL·kg^−1^·min^−1^, corresponding to ~43.7 ± 11.04% of VO_2_max [5]. However, the available evidence shows considerable heterogeneity, likely due to the limited number of studies and the diversity of the samples analysed. In this regard, VO_2_max values of 51.15 ± 5.73 mL·kg^−1^·min^−1^ have been reported in amateur players [9], whereas higher values of 55.43 ± 7.04 mL·kg^−1^·min^−1^ have been observed in professional players [10]. Regarding neuromuscular responses, competition does not appear to induce substantial levels of fatigue, as variables such as jump height and handgrip strength [11] generally do not show significant post-match declines. This limited and heterogeneous body of evidence highlights the need for further investigation of the physical and physiological characteristics of padel players, not only to accurately describe their demands, but also to establish reference values that facilitate training optimisation and the individualisation of interventions based on variables such as weekly training frequency or playing side.

With regard to the playing side, a defining characteristic of padel is the high degree of positional specialization. In contrast to other racket sports that require frequent side rotation, padel players generally maintain a fixed side assignment (either left or right) throughout the match. Previous literature has reported relevant differences primarily from a technical–tactical perspective. In terms of playing volume, left-side players are more involved in match play, performing a greater total number of strokes [12,13]. Concerning stroke type and court location, left-side players execute a higher number of strokes in intermediate court areas [14,15] and at the net [15], whereas right-side players perform more smashes from both net and baseline positions [14]. From the baseline, no significant differences have been observed in the number of lobs; however, left-side players tend to execute more parallel shots, while right-side players perform more cross-court shots [16]. In addition, right-side players show a higher frequency of bajadas (i.e., an offensive shot in which the ball bounces on the ground and then on the glass [17]) [18] and back wall shots [19]. In the net and mid-court zones, right-side players perform a greater number of volleys [12], whereas left-side players execute more overhead shots, such as the smash, including both winners and unforced errors [20,21]. Collectively, these patterns suggest that right-side players tend to prioritise rally continuity, while left-side players assume a more offensive role [22,23]. Beyond their technical–tactical relevance, these differentiated roles may have important physical and physiological implications. A more offensive profile, characterised by frequent smashes, net approaches, and point-ending actions, likely entails a greater number of explosive movements, rapid accelerations, and vertical jumps. In contrast, a role focused on rally construction and continuity may involve longer exchanges, repeated submaximal efforts, defensive transitions, and efficient recovery between actions. Consequently, the tactical responsibilities associated with each playing side may translate into distinct external loads, acute physiological responses, and long-term neuromuscular and metabolic adaptations. Thus, playing side not only shapes technical–tactical behaviour but may also modulate the physical demands imposed on players during match play.

From a physical perspective, previous studies have shown contradictory findings regarding side-specific profiles. Interestingly, while the offensive nature of the left-side position would theoretically suggest superior explosive capacity, some research has indicated that right-side players exhibit greater jump height in CMJ and squat jump (SJ) tests, whereas left-side players present higher muscle mass and maximal strength [24]. This reversed expectation—where the theoretically less ‘explosive’ role demonstrates higher vertical jump values—highlights a gap in the understanding of how tactical roles translate into physical status. However, when competition-related demands have been analysed, left-side players have been shown to perform a greater number of accelerations and decelerations per hour compared with right-side players [25], as well as a greater post-match increase in handgrip strength [26]. From a physiological standpoint, competition does not appear to significantly affect cytokine responses [27], nor biochemical markers such as serum creatinine, urea, and creatine kinase (CK), or urinary parameters including specific gravity and erythrocyte count [28]. Although some evidence suggests slight differences according to playing side [24], the limited and heterogeneous nature of the available data restricts meaningful comparisons across studies, particularly when considering sex-related differences. This limitation highlights the need to generate further evidence on physical fitness and physiological parameters in padel according to playing side, particularly considering that the differentiated tactical functions of each position could theoretically promote specific adaptations, such as greater explosive power and anaerobic contribution in left-side players, and enhanced repeated-effort capacity and recovery efficiency in right-side players.

Overall, the existing literature underscores the need to further investigate the physical and physiological characteristics of padel players, given the limited research currently available in this area [8]. Moreover, differences associated with playing side, together with potential disparities in workload, may result in distinct physical and physiological adaptations, as the specific technical–tactical roles performed during competition may expose players to different frequencies and intensities of explosive actions, accelerations, decelerations, and recovery periods. Weekly training frequency may further modulate these adaptations, particularly in high-level players. Therefore, the aim of the present study was to analyse physical and physiological differences in male padel players according to playing side. A secondary objective was to observe the influence of training volume on these parameters. It was hypothesised that left-side players presented higher values in physical fitness, particularly in neuromuscular performance-related variables. The secondary hypothesis was that high-load players would present higher physical fitness values than low-load players.

## 2. Materials and Methods

### 2.1. Study Design

This study employed a cross-sectional, observational design to examine differences in physical performance among padel players according to playing side and weekly training frequency. Although group comparisons were conducted, the study was not quasi-experimental, as no independent variables were manipulated and no participants were assigned to specific conditions. Playing side and weekly training frequency represented pre-existing characteristics related to each player’s competitive role and habitual training practice; therefore, experimental allocation was neither feasible nor ethically appropriate. Accordingly, the design aimed to identify naturally occurring group differences rather than establish causal relationships.

Data collection was conducted in May under controlled environmental conditions (mean temperature: 15.2 °C; relative humidity: 42%). All participants were informed about the study objectives and procedures and provided written informed consent in accordance with the Declaration of Helsinki [29]. The study protocol was approved by the Ethics Committee of the University of Extremadura (166//2023; approved 15 December 2023).

To minimise the potential influence of residual fatigue, assessments were administered in a standardised order: (i) anthropometric measurements; (ii) vertical jump performance (CMJ and ABK tests); (iii) isometric handgrip strength; (iv) agility (*t*-test); and (v) intermittent endurance (Yo-Yo Intermittent Recovery Test Level 2). Standardising the testing sequence ensured internal consistency across participants while maintaining the strictly observational nature of the study.

### 2.2. Participants

The sample comprised 14 high-level male padel players, aged 18–34 years, who were actively competing in professional circuits or top-level regional federated competitions. Specifically, all participants were ranked within the top 10 positions of their respective regional federations in Spain. Players were excluded if they had sustained an injury within the three months preceding data collection or were not at full physical capacity at the time of testing. Participants were contacted by telephone to schedule the testing sessions. Based on their habitual playing role in competition, players were classified according to playing side, resulting in two groups: left-side players (*n* = 7; age: 25.14 ± 5.01 years; height: 1.82 ± 0.05 m; body mass: 78.86 ± 4.98 kg; weekly training volume: 11.50 ± 3.84 h) and right-side players (*n* = 7; age: 20.86 ± 2.04 years; height: 1.77 ± 0.05 m; body mass: 73.00 ± 5.26 kg; weekly training volume: 12.86 ± 3.81 h). Comparisons of baseline characteristics showed trends toward greater height and body mass in left-side players; however, these differences did not reach statistical significance (height: *t* = −2.010, df = 12, *p* = 0.068; body mass: *t* = −2.139, df = 12, *p* = 0.054; Cohen’s *d* = −1.074 and −1.143, respectively). Additionally, players were categorised according to weekly playing frequency into low-frequency (*n* = 7; <10 h·week^−1^) and high-frequency (*n* = 7; >10 h·week^−1^) groups. Comparisons between frequency groups revealed no significant differences in height or body mass (height: *t* = −1.482, df = 12, *p* = 0.164; body mass: *t* = −0.133, df = 12, *p* = 0.896; Cohen’s *d* = −0.792 and −0.071, respectively). The 10 h threshold corresponded to the sample’s mean weekly playing time, providing a data-driven criterion for group classification. This approach allowed the formation of two balanced groups reflecting the cohort’s central tendency and facilitated statistical comparison without relying on an arbitrary or literature-based cut-off.

### 2.3. Procedures

The study was conducted across two separate Mondays, one week apart, with both sessions starting at 9:00 a.m. The first session was dedicated to familiarisation with the testing procedures, while the second session corresponded to definitive data collection. During the familiarisation session, participants received standardized instructions and performed two to three submaximal practice trials for each test to ensure correct technique and understanding of the procedures. Verbal feedback was provided when necessary to standardize movement execution and reduce potential learning effects during the experimental session. All assessments were performed during a period in which none of the participants had competed during the preceding weekend, thereby minimising the influence of residual fatigue and ensuring homogeneous testing conditions. In addition, testing was conducted during the competitive season to avoid potential performance fluctuations associated with preseason or end-of-season phases.

Upon arrival at the testing facility, participants completed a descriptive questionnaire and underwent an anthropometric assessment. Body mass and total height were measured, and, subsequently, the length of the lower limbs was measured from the iliac crest. Prior to testing, participants performed a standardised 10 min warm-up consisting of low-intensity jogging and dynamic locomotor exercises, followed by dynamic joint mobility drills. The protocol concluded with submaximal countermovement jumps, short accelerations, and lateral displacements to ensure neuromuscular readiness without inducing fatigue. After the warm-up, the following physical performance tests were administered.

Vertical Jump Performance: Vertical jump height was assessed using the My Jump Lab 3 application (Madrid, Spain) [30], which has been previously validated for this purpose. Recordings were obtained using an iPhone 16 Pro Max (Apple Inc., Cupertino, CA, USA), utilising the rear camera at 240 frames per second in slow-motion mode to ensure accurate frame-by-frame identification of take-off and landing. Two jump conditions were evaluated: the CMJ and the ABK. For the CMJ, participants were strictly required to keep their hands on their hips throughout the entire movement (akimbo position) to isolate lower-limb explosive power and eliminate any momentum from arm swing. The CMJ was initiated from an upright standing position, followed by a rapid downward movement immediately preceding the concentric propulsion phase until take-off [31]. The ABK was performed using the same technique, but it was executed with a free arm swing to generate additional impulse [32]. Each jump condition was performed three times, with 20 s of rest between attempts. The mean value of the three trials was used for analysis. A 1 min recovery period was allowed between jump types, and 2 min of rest were provided between the final CMJ attempt and the first ABK attempt.

Isometric Handgrip Strength: Isometric handgrip strength was assessed using a Takei handgrip dynamometer (Takei, Tokyo, Japan), a device widely used in sport science research [33]. Following established protocols [34], the participants stood upright with feet shoulder-width apart, the testing arm fully extended alongside the body, and the grip held in a neutral position. Prior to data collection, the dynamometer was calibrated according to the manufacturer’s instructions to ensure measurement accuracy. The grip span was individually adjusted to each participant’s hand size, ensuring that the proximal interphalangeal joint of the fingers was positioned at approximately 90° during contraction. Three maximal attempts were performed with each hand, and the mean value was retained for analysis. All measurements were conducted by the same experienced evaluator to minimise inter-tester variability. A 1 min rest period was allowed between attempts, and 2 min of rest were provided between testing hands.

*t*-Test: Test used to evaluate the agility and change-of-direction speed, following the protocol described by Hernández-Davó et al. [35]. A central cone was placed 10 m from the timing gates, with two additional cones positioned 5 m to each side of the central cone. On the starting signal, participants sprinted forward through the timing gates (Witty System, Microgate, Bolzano, Italy) and touched the central cone with the right hand. They then performed a 5 m lateral shuffle to the left to touch the left cone with the left hand, followed by a 10 m lateral shuffle to the right to touch the right cone with the right hand. Subsequently, participants shuffled laterally 5 m back to the central cone, executed a 180° turn, and sprinted forward 10 m to complete the test. Each participant performed three trials, with 2 min of passive recovery between trials. The best time was used for statistical analysis.

Consistent with established protocols for agility and change-of-direction speed assessments, the best performance (fastest time) among the three trials was retained for analysis. This approach is preferred in maximal speed tasks to represent the athlete’s peak anaerobic and neuromuscular potential, whereas the mean value was utilized for jump and strength tests to ensure a more stable and reliable measure of consistent force production.

Yo-Yo Intermittent Recovery Test level 2: Test used to assess the Intermittent endurance capacity, which has previously been applied in padel players [36]. The protocol described by Bangsbo et al. [37] was followed, with a maximum test duration of 15 min. The initial running speed was set at 13 km·h^−1^ and increased progressively according to audio signals. The test consisted of repeated shuttle runs over a 20 m distance, marked by two cones, combined with a 5 m active recovery zone indicated by a third cone. Each cycle comprised a 20 m out-and-back run (40 m) followed by an out-and-back active recovery run (10 m), for a total distance of 50 m per cycle. The test was terminated when the participant failed to reach the running zone within the required time on two consecutive occasions. Maximal oxygen uptake (VO_2_max) was estimated from the total distance covered using the following equation: VO_2_max (mL/kg/min) = YoYo IR2 distance (m) × 0.0136 + 45.3 [37]. It should be noted that this equation provides an indirect estimation of VO_2_max derived from field-based intermittent performance rather than direct laboratory gas analysis. Therefore, the reported VO_2_max values should be interpreted as approximations of aerobic capacity and indicators of intermittent endurance performance, acknowledging the potential estimation error compared to gold-standard cardiopulmonary assessment.

### 2.4. Statistical Analysis

Results are presented as mean ± standard deviation. Data were assessed for normality and homogeneity of variance using the Shapiro–Wilk and Levene’s tests, respectively. Descriptive analysis includes mean ± standard deviation, minimum and maximum, 95% confidence interval mean, coefficient of variation (CV) and 25th, 50th and 75th percentiles. CV values < 10% and <15% considered to represent excellent and acceptable consistency, respectively, in accordance with established sports science criteria. These thresholds provide a standardized framework to interpret the reliability and inter-subject variability of the physical performance metrics evaluated. Paired-samples *t*-tests were used to compare vertical jump height and isometric strength. Independent-samples Student’s *t*-tests were applied to compare players based on playing side and weekly training frequency for variables with normal distribution and equal variances. As the study hypotheses were explicitly directional and formulated a priori—anticipating higher physical performance values in left-side players and in players with greater weekly training frequency—one-tailed *t*-tests were applied to test these specific hypotheses. The decision to employ one-tailed analyses was established prior to data collection and was theoretically grounded in the framework presented in the introduction, which proposed directional superiority patterns rather than non-directional differences. To enhance transparency and allow interpretation from a more conservative perspective, exact *p*-values, effect sizes, and the direction of observed differences are reported, enabling readers to interpret findings within both directional and non-directional contexts. Effect sizes for pairwise differences were calculated using Cohen’s *d*, with the following qualitative interpretation: *d* < 0.2 (no effect), 0.2–0.49 (small effect), 0.5–0.79 (moderate effect), and ≥0.8 (large effect) [38]. All statistical analyses were conducted using JASP for Windows (version 0.19, Amsterdam, The Netherlands). Statistical significance was set at *p* < 0.05.

## 3. Results

Table 1 presents the physical performance characteristics of the players. Vertical jump height was significantly greater in the ABK compared with the CMJ (df = 13, *t* = −10.148; *p* < 0.001; Cohen’s *d* = 0.160). Isometric handgrip strength showed significant inter-limb differences (df = 13, *t* = 7.304; *p* < 0.001; Cohen’s *d* = 0.230), with higher values observed in the dominant hand. Mean *t*-test completion time was 10.40 s (95% CI: 10.06–10.74 s). In the Yo-Yo test, players covered a mean distance of 404.28 m, corresponding to an estimated VO_2_max of 50.79 (95% CI: 50.02–51.57 mL·kg^−1^·min^−1^).

**Figure 1:**
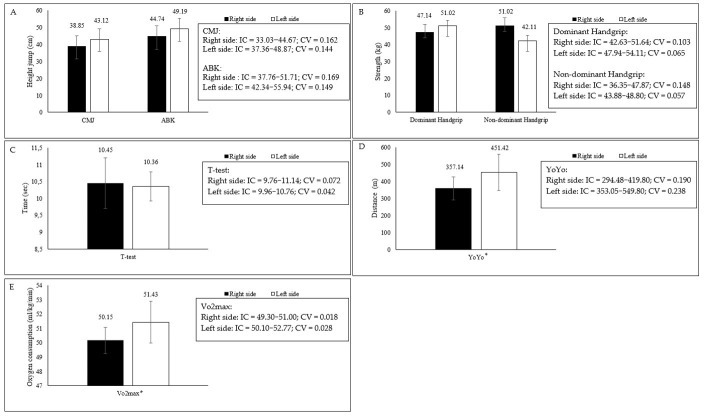
Comparative analysis according to the side of play. Note. Comparison between groups in physical performance variables: (**A**) jump performance; (**B**) isometric strength; (**C**) agility; (**D**) endurance test; and (**E**) maximal aerobic capacity. Data are presented as mean ± SD. * indicates statistically significant differences between groups (*p* < 0.05).

Playing side exerted a significant effect on Yo-Yo test performance and estimated VO_2_max (*t* = −1.978; df = 12; *p* = 0.036; Cohen’s *d* = −1.057) (Figure 1), being higher in left-side players. Although large effect sizes were observed for handgrip strength favoring left-side players, these differences did not reach statistical significance (*p* > 0.05). Given the limited statistical power, these findings should be interpreted as exploratory trends.

Weekly training frequency (Table 2) revealed no statistically significant differences between the high-load and low-load groups for any of the analyzed performance variables (*p* = 0.198–0.608). Specifically, trivial to small effect sizes were observed for CMJ (*p* = 0.443; *d* = 0.078), ABK (*p* = 0.442; *d* = 0.08), maximal handgrip strength (*p* = 0.478; *d* = 0.031), normalized handgrip strength (*p* = 0.441; *d* = 0.081), Yo-Yo test performance (*p* = 0.601; *d* = −0.14), and estimated VO_2_max (*p* = 0.601; *d* = −0.14), indicating minimal practical differences between training-load groups. In contrast, the *t*-test demonstrated a small effect size despite the absence of statistical significance (*p* = 0.198; *d* = 0.47). The low-load group achieved faster times (10.26 ± 0.38 s) compared with the high-load group (10.54 ± 0.75 s) and also displayed a substantially lower coefficient of variation (CV = 0.037 vs. 0.071).

## 4. Discussion

The aim of the present study was to analyse physical and physiological differences in male padel players according to playing side and weekly training frequency. The main findings indicate that: (i) players displayed significantly lower vertical jump performance in the CMJ compared with the ABK (40.98 vs. 46.96 cm), while isometric handgrip strength was greater in the dominant than in the non-dominant hand (49.08 vs. 44.22 kg); additionally, mean values obtained in the *t*-test (10.40 s), Yo-Yo test level 2 (404.28 m), and estimated VO_2_max (50.79 mL·kg^−1^·min^−1^) characterize the change-of-direction ability and aerobic capacity of high-level padel players; (ii) playing side significantly influenced Yo-Yo test performance and VO_2_max, with higher values observed in left-side players, whereas no statistically significant differences were detected for handgrip strength despite large effect sizes and low variability; and (iii) weekly training frequency did not result in statistically significant differences in any physical variable, although agility performance showed a small effect size, favouring the low-load group.

The physical performance values reported in this study provide updated reference data that help to define the physical profile of competitive padel players. Regarding vertical jump performance, the CMJ values observed (~41 cm) exceed those previously reported in professional (≈32 cm) [10], amateur (≈34.6 cm) [36], female (≈24 cm) [10], and youth padel players (26–30 cm) [39,40,41]. Although both our study and previous research [10,36] followed a standardized protocol with hands placed on the hips to eliminate arm-swing momentum, certain methodological nuances should be considered. Our results were obtained using the My Jump Lab app, which calculates jump height through flight time via high-speed video, whereas earlier studies [10,36] primarily used a contact platform. While both methods are validated, slight discrepancies in landing mechanics or flight-time estimation can occur. Furthermore, the differences across competitive levels and age groups suggest that neuromuscular performance in padel does not increase linearly with playing level and may be influenced by training background, playing role, or evolving game demands. Consequently, further research is needed to establish contemporary reference values and support the individualization of training strategies.

ABK performance (~46 cm) was lower than values typically reported in sports such as basketball or volleyball [42,43], where arm swing plays a more decisive role due to sport-specific demands. However, such cross-sport comparisons should be interpreted cautiously, as vertical jump capacity serves distinct functional purposes depending on the sport. In basketball and volleyball, jump performance constitutes a primary competitive requirement, whereas in padel it represents a complementary neuromuscular quality rather than a decisive match-performance factor. Conversely, ABK values were comparable to those reported in racket sports such as tennis [42], where external load and movement patterns, particularly in doubles formats, are similar to those observed in padel [44]. Notably, when compared with previous studies in professional padel players [10], the values obtained in the present investigation were higher, supporting the notion that the physical demands of padel may have increased in recent years and reinforcing the need for updated research reflecting the current performance profile of the sport.

Isometric handgrip strength results revealed clear asymmetries between the dominant and non-dominant limbs, which are likely attributable to the unilateral nature of racket use. Such asymmetries have been consistently reported in padel and other racket sports [45,46]. Although functional asymmetries are inherent to unilateral sports and do not necessarily imply an increased injury risk [47], pronounced asymmetries have been associated with a greater likelihood of injury in some contexts [48]. Specifically, inter-limb differences exceeding 10–15% have been suggested as a threshold beyond which injury risk may increase [48]. Therefore, monitoring handgrip strength asymmetry remains relevant for injury prevention and compensatory training strategies.

Agility and change-of-direction ability, assessed using the *t*-test, yielded mean values (~10.40 s) consistent with the multidirectional demands of padel. These results compare favourably with those reported in young tennis players (~10.68 s) [35], volleyball female and basketball male players (~12 s) [49,50], while remaining higher than values observed in elite badminton players (~9.14 s) [51]. Given the high frequency of accelerations, decelerations, and directional changes during padel match play [52], these findings underscore agility as a key physical quality in this sport and support its systematic assessment and targeted development in training programmes.

Performance in the Yo-Yo Intermittent Recovery Test revealed a mean distance of 404.28 m and an estimated VO_2_max of 50.79 mL·kg^−1^·min^−1^. These values fall within the range previously reported in laboratory-based assessments of padel players (43.2–59.4 mL·kg^−1^·min^−1^) [5], thereby contributing to the limited body of evidence on aerobic capacity in this sport. From a comparative perspective, the VO_2_max observed in padel players can be considered moderate, lower than values reported in badminton, yet higher than those observed in combat sports (e.g., judo or taekwondo) and certain team sports such as rugby or basketball [53]. This aerobic profile appears sufficient to support recovery between points, maintain match intensity, and sustain performance during prolonged rallies.

Regarding playing side, despite the initial hypothesis anticipating greater physical advantages in left-side players, based on previous evidence suggesting that left-side players typically assume more offensive roles, participate more frequently in point-ending actions (e.g., smashes) [12,13,14], and may therefore be exposed to higher neuromuscular and explosive demands during match play. However, the results demonstrated significant differences only in Yo-Yo test performance and VO_2_max, favouring left-side players. Our findings suggest that positional specialization in elite padel requires differentiated physical conditioning programs. Given that left-side players exhibit a higher intermittent endurance capacity and a greater demand for upper-body explosive power for finishing actions (smashes), strength and conditioning coaches should prioritize intermittent High-Intensity Interval Training (HIIT) protocols and upper-limb power exercises for these left-side padel players. On the other hand, no statistically significant differences were observed in jump performance or agility, as observed in previous studies [54,55], although conflicting findings have been reported elsewhere [24]. These discrepancies may be attributed to differences in competitive level, sample characteristics, or the specific technical–tactical demands associated with each playing position.

Change-of-direction performance did not differ significantly between playing sides, suggesting that agility demands are relatively symmetrical, as both players are exposed to frequent multidirectional movements during match play [24,55]. In contrast, isometric handgrip strength tended to be higher in left-side players, aligning with findings in amateur padel players [55] and partially supporting the proposed hypothesis. This difference may reflect the greater involvement of left-side players in point-ending actions [56], particularly smashes [17], which require higher stroke velocities and force production [57]. Consequently, handgrip strength may reflect specific neuromuscular adaptations linked to the tactical role performed on court.

One of the most novel findings of the present study is the higher VO_2_max observed in left-side players, which is consistent with both the initial hypothesis and previous reports favoring this playing position [24]. From a technical–tactical perspective, right-side players tend to be more involved in rally continuity actions [58], such as the bandeja (i.e., aerial shot similar to the forehand volley, with an impact range of 1:30 to 3 o’clock [17]) and the backhand volley [23], which may require sustained movements and frequent positional adjustments. However, left-side players appear to be exposed to a greater external load, characterized by a significantly higher number of accelerations and decelerations, along with slightly greater total distance covered during match play [25]. Although external load was not directly measured in the present study, these previously reported positional differences could hypothetically contribute to distinct aerobic profiles. Therefore, the proposed link between greater movement demands and higher VO_2_max values in left-side players should be considered hypothesis-generating rather than confirmatory. Future studies integrating physiological testing with direct external load monitoring are warranted to clarify this relationship.

Finally, weekly training frequency did not significantly influence any of the physical capacities assessed. Although higher training volumes are generally associated with improved physical fitness [59], no differences were observed between players with higher and lower weekly padel practice in this cross-sectional analysis. These findings do not provide evidence that greater on-court practice volume alone is associated with superior strength, power, or aerobic capacity within this sample. Several factors may explain this outcome: training frequency does not necessarily reflect quality, intensity, or specific content; technical–tactical work may have predominated over structured conditioning; and inter-individual variability or complementary off-court training could have influenced results. Given the importance of vertical jump performance and agility for padel success [60,61], practitioners should consider integrating targeted strength and conditioning interventions. Based on the physical profile observed—particularly the relevance of lower-limb explosive performance and inter-limb asymmetries—recommended strategies include multi-joint lower-body resistance exercises (e.g., squats, lunges), unilateral strength work, plyometric training to enhance stretch–shortening cycle efficiency, and high-intensity interval training to support aerobic capacity. Such interventions may enhance performance [62], and contribute to injury prevention [63], although the latter was not directly examined in this study.

The present study has several limitations that should be considered when interpreting the findings. First, the relatively small sample size limits the generalizability of the results. To further contextualize this limitation, a post hoc power analysis was conducted for the primary between-group comparison (Yo-Yo test performance), using the observed effect size (*d* = 1.057), α = 0.05, and *n* = 7 per group. The analysis indicated an achieved statistical power of 58.68%, suggesting that the study was sufficiently powered to detect large effects but underpowered to reliably identify small-to-moderate differences. This reinforces the need for cautious interpretation of non-significant findings.

Although players were categorised according to playing side and weekly training frequency, group allocation was not randomised. This may have resulted in partial group imbalance, with unequal group sizes potentially affecting statistical power and reducing sensitivity to detect small-to-moderate effects. Therefore, the absence of significant differences in certain variables should not be interpreted as definitive evidence of equivalence between groups, but rather considered in light of possible type II error.

Second, although all assessment instruments employed have been previously validated, the use of more specific measurement tools, such as force platforms for vertical jump assessment, could have provided additional biomechanical variables (e.g., impulse, peak force, or rate of force development). The inclusion of such measures would allow a more detailed examination of jump strategies and potential inter-individual or positional differences.

In addition, logistical constraints limited the number of assessments performed, restricting the analysis primarily to descriptive data obtained at a single time point. While this approach allowed for the characterization of the players’ physical profile, it did not permit the investigation of medium- or long-term adaptations to training or competition. Given the cross-sectional nature of the design, the observed differences between groups should be interpreted as associations rather than evidence of causal relationships. Consequently, it cannot be determined whether playing side or weekly training frequency directly contributed to specific physical characteristics, nor whether these characteristics represent true training adaptations. Therefore, future research should aim to include larger samples, adopt longitudinal study designs, and integrate more comprehensive methodologies, such as external load monitoring and advanced neuromuscular assessments, to further elucidate the physical and physiological demands of padel performance.

## 5. Conclusions

The present study provides relevant evidence regarding the physical characteristics of high-level padel players, as well as potential differences according to playing side and weekly training frequency. The descriptive values obtained for vertical jump performance, with mean heights of 40.98 cm in the CMJ and 46.96 cm in the ABK, appear to be representative of the physical condition of the players analyzed. These values offer preliminary reference data for practitioners working with similar competitive populations; however, due to the relatively small sample size, they should be interpreted as exploratory benchmarks rather than definitive normative standards. No direct relationships with match performance were examined.

With respect to isometric handgrip strength, a clear asymmetry between the dominant and non-dominant limbs was observed, with higher values in the dominant arm. Although such asymmetry does not necessarily imply an immediate negative impact on performance or injury risk, it represents a relevant characteristic in unilateral sports and should be monitored to prevent excessive muscular imbalances over time.

The agility and change-of-direction performance assessed using the *t*-test (mean time: 10.40 s) reflects an adequate displacement capacity in this cohort. Similarly, the mean distance covered in the Yo-Yo Intermittent Recovery Test level 2 (404.28 m), corresponding to an estimated VO_2_max of 50.79 mL·kg^−1^·min^−1^, indicates a good level of intermittent endurance capacity in the players assessed. These findings describe the physical profile of the sample but should not be interpreted as direct determinants of competitive success.

Regarding the playing side, left-side players exhibited greater isometric handgrip strength and higher estimated VO_2_max values. These differences should be interpreted as positional associations observed within this sample rather than evidence of specific physiological adaptations caused by tactical role. Although tactical demands may contribute to distinct physical profiles, the cross-sectional design does not allow causal inferences.

Finally, while weekly padel training frequency was not associated with significant differences in the variables assessed, this finding should be interpreted cautiously given the sample size and observational nature of the study, which may have reduced statistical power and sensitivity to detect small-to-moderate effects.

From a practical standpoint, the results suggest that complementary strength and conditioning strategies may be beneficial to support the physical demands of high-level padel. However, such recommendations are derived from general training principles and the observed physical profile of the players, rather than from direct intervention evidence within this study. Therefore, future experimental research is needed to determine the effectiveness of specific training interventions in improving padel performance.

Future research should confirm these findings in larger, more diverse cohorts, adopt longitudinal designs to assess chronic adaptations, and integrate external load monitoring with performance assessments to better understand the interplay between tactical role, training load, and physical development.

## Figures and Tables

**Table 1:** Descriptive analysis of the physical variables.

				95% Confidence Interval Mean			Percentile		
Variable	Mean	Std. Deviation	Minimum–Maximum	Upper	Lower	CV	25th	50th	75th
CMJ (cm)	40.98	6.40	28.03–53.20	37.29	44.68	0.156	36.54	42.32	44.55
Abalakov (cm)	46.96	7.49	34.07–62.83	42.63	51.29	0.160	41.16	47.45	51.67
Dominant Handgrip (kg)	49.08	4.49	39.40–54.50	46.49	51.67	0.091	47.32	50.45	52.35
Non-dominant Handgrip (kg)	44.22	5.09	33.90–50.20	41.28	47.17	0.115	43.20	45.65	47.47
*t*-test (s)	10.40	0.59	9.53–11.96	10.06	10.74	0.057	10.18	10.38	10.64
YoYo (m)	404.28	98.66	260.00–620.00	347.32	461.25	0.244	330.00	390.00	455.00
VO_2_max (mL/kg/min)	50.79	1.34	48.83–53.73	50.02	51.57	0.026	49.78	50.60	51.48

Note. CV: coefficient of variation.

**Table 2:** Comparative analysis according to the weekly training frequency.

	Group	Mean	Std. Deviation	SE	IC	CV	*p*	Cohen’s *d*
CMJ (cm)	High-load	41.24	6.89	2.608	34.86–47.62	0.167	0.443	0.078
	Low-load	40.73	6.41	2.426	34.79–46.66	0.158		
Abalakov (cm)	High-load	47.27	6.70	2.535	41.07–53.48	0.142	0.442	0.080
	Low-load	46.65	8.75	3.308	38.56–54.75	0.188		
Dominant Handgrip (kg)	High-load	49.15	3.88	1.467	45.56–52.74	0.079	0.478	0.031
	Low-load	49.01	5.34	2.021	44.06–53.96	0.109		
Non-dominant Handgrip (kg)	High-load	44.44	5.67	2.146	39.19–49.69	0.128	0.441	0.081
	Low-load	44.01	4.89	1.849	39.48–48.53	0.111		
*t*-test (s)	High-load	10.54	0.75	0.284	9.85–11.24	0.071	0.198	0.470
	Low-load	10.26	0.38	0.144	9.91–10.62	0.037		
YoYo (m)	High-load	397.14	95.51	36.103	308.80–485.48	0.241	0.601	−0.140
	Low-load	411.42	108.84	41.140	310.76–512.09	0.265		
VO_2_max (mL/kg/min)	High-load	50.70	1.29	0.491	49.50–51.90	0.026	0.601	−0.140
	Low-load	50.89	1.48	0.560	49.52–52.26	0.029		

Note. For all tests, the alternative hypothesis specifies that group High-load is greater than group Low-load. IC: interval confidence; CV: coefficient of variation; SE: Standard error; *p*: significance; negative values of Cohen’s *d* indicate higher mean values in the Left group compared with the Right group, consistent with the specified directional hypothesis.

## Data Availability

The original contributions presented in this study are included in the article. Further inquiries can be directed to the corresponding author.
